# Whole Coffee Cherry Extract Improves Working Memory and Response Inhibition: Acute and Longitudinal Results from a Remote, Randomized, Double-Blind, Placebo-Controlled Clinical Trial

**DOI:** 10.3390/nu16142348

**Published:** 2024-07-20

**Authors:** Jennifer L. Robinson, John M. Hunter, Megan Kern, Merlina Rodas, Jasmine Jowers, Jenna Robertson, Caitlyn Wanalista

**Affiliations:** 1 Department of Psychological Sciences, Auburn University, Auburn, AL 36849, USA; 2 Auburn University Neuroimaging Center, Auburn University, Auburn, AL 36849, USA; 3 VDF FutureCeuticals, Inc., Momence, IL 60954, USA

**Keywords:** polyphenols, cognition, brain health, aging, nutraceuticals

## Abstract

Earlier laboratory-based evidence has suggested that polyphenol-rich, decaffeinated whole coffee cherry extract (CCE) supports improvements in acute and long-term cognitive performance. To better understand CCE’s potential to promote cognitive processing, we conducted a first-of-its-kind remote clinical trial. Participants were randomized into one of two intervention arms: placebo or 200 mg CCE. At the beginning of the study, participants were asked to complete a set of acute cognitive challenges as part of the baseline assessment. Tasks were nearly identical to those used in previous, laboratory-based research. Acute results support that CCE outperformed placebo, reducing omissions and improving accuracy, during working memory and inhibitory control tasks. Long-term results indicate that CCE outperformed placebo on a measure of accuracy. This contributes to the literature in three ways: (1) results improve upon previously reported robust and consistent findings in a real-world setting that a single-dose of CCE acutely improved cognitive performance; (2) results replicate previous laboratory findings but in a real-world setting that long-term CCE supplementation outperformed placebo on measures of accuracy in a working memory task; and (3) it serves as proof of concept of a novel remote clinical trial model that may provide real-world evidence of efficacy while increasing accessibility and cohort diversity.

## 1. Introduction

There has been increasing interest in natural dietary supplements that may support healthy cognition. Among their many reported health benefits, preliminary evidence suggests that bioactive phytochemicals (e.g., polyphenols) may improve cognition, particularly in aging populations [1,2,3]. However, few studies have assessed whether these outcomes generalize to real-world settings.

Whole coffee cherry extract (CCE) is a proprietary, safe, powdered extract of whole coffee cherries from *Coffea arabica* with high levels of coffee polyphenols (for information on the quantification of polyphenols, please see [4,5,6,7]) and substantially low (<2%; <4 mg/dose) levels of caffeine. The high-polyphenol CCE material is produced by VDF FutureCeuticals, Inc. (Momence, IN, USA) (U.S. patent 7,815,959) using an ethanol/water food grade extraction protocol according to a proprietary process that assures adherence to carefully established specifications and standards. The CCE extract has been produced from several varieties of *Coffea arabica* plants, with most recent sourcing from dedicated plantations in India. Decaffeination occurs as part of the production process for CCE. All batches of CCE are tested to ensure caffeine levels are negligible. CCE has been previously associated with increased serum concentrations of both circulating and exosomal brain-derived neurotrophic factor (BDNF), in addition to increased alertness and decreased fatigue [8,9] (please see U.S. patents 11,471,500 and 12,036,261). Mechanistically, BDNF represents one of several potential pathways through which CCE may support or even enhance brain health and/or function. Pre-clinical research has suggested that polyphenols, and coffee cherry products in particular, decrease inflammation through modulation of specific targets, and may also work through gut–brain-axis-dependent mechanisms [10]. These mechanisms, along with evidence from other organ systems, provide plausibility that CCE could contribute to improvements in brain function.

Despite the mounting evidence, additional well-powered and well-designed studies are needed to more clearly understand the effects of CCE. Preliminary and pilot studies have demonstrated that CCE may improve several measures of cognition in older adults (i.e., 55–65 years of age) exhibiting mild cognitive decline, and that these improvements may be driven by distinct neurophysiological changes [5,11,12]. For example, our lab found that a single acute 100 mg dose of CCE led to rapid changes in brain networks associated with working memory concomitant with improvements in performance on a common working memory task (*n*-back) and a response inhibition task (Go/No-Go). Another, larger cohort study demonstrated longer-term effects at 100 mg and 200 mg total daily CCE dosages that emerged as soon as after 7 days and persisted over 28 days [12].

Interestingly, all studies to date have been conducted in sterile, traditional laboratory environments, which by definition do not automatically lend themselves to real-world generalizability. Indeed, there has been recent discussion in the literature related to the deficiencies of traditional clinical models [13,14,15]. Accordingly, we designed and conducted a first-of-its-kind remote clinical trial to assess both the acute and long-term effects of CCE. We leveraged a double-blind, randomized, placebo-controlled, two-arm design (i.e., placebo versus CCE). Participants were recruited from across the United States. Although the study was conducted for 28 days, of particular initial interest was whether or not the acute results from our previous study would replicate in a real-world environment. Consequently, at the beginning of the long-term study, we required the participants to perform a pre- (baseline) assessment and a single-dose, acute (one-hour post-administration) cognitive assessment. We hypothesized that the CCE group, after a single dosage of 200 mg, would show improved performance post-administration on both the working memory (i.e., that which lies between immediate recall and long-term memory and has as a primary feature the active use or processing of memory to reason, problem solve, and plan) and the inhibitory control tasks (i.e., one’s ability to refrain from responding to an automatic or prepotent stimulus), as reported in previous research studies. Furthermore, we hypothesized that the CCE group would show greater improvements over the course of the 28-day supplementation period. If true, the results would provide robust evidence for distinct cognitive improvements outside of a laboratory environment.

## 2. Materials and Methods

### 2.1. Study Design

We conducted a double-blind, randomized, placebo-controlled remote clinical trial (https://clinicaltrials.gov/study/NCT04986956), approved by the Auburn University Institutional Review Board. Participants were given either 200 mg CCE or a microcellulose placebo, both of which are generally-recognized-as-safe (GRAS) materials, to take every day for 28 days upon waking. Every 7 days, participants took a cognitive assessment via the internet using the Millisecond—Inquisit (Version 6.5.1; Seattle, WA, USA) platform [16].

On the first day of the study, participants completed the cognitive assessment before, and 1 h after, taking their study material (i.e., the “acute cognitive challenge”). Thereafter, on subsequent cognitive assessment days (7, 14, 21, and 28), participants were asked to refrain from taking their study material until after the assessment was completed in order to assess any cumulative effects of treatment. Stated differently, we wanted to be able to assess any accumulated changes in their baseline cognitive behaviors without any potential influence from acute exposure to the study supplements, since we hypothesized that CCE might materially impact study subjects both acutely and non-acutely.

The cognitive assessment was composed of tasks that broadly tested working memory, focus, attention, accuracy, and response inhibition. The overall cognitive assessment was composed of the following tasks (in order of delivery): (1) *n*-back [5,17,18,19], (2) a face-name paired associate task [20,21], (3) a Stroop task [22,23], (4) a Go/No-Go task [5,24], (5) an attention network task [25], (6) a symbol search task [26,27], (7) a trail-making task [28,29], (8) a face-in-the-crowd task [30], and finally (9) a repetition of the *n*-back task. Breaks were incorporated into the assessment.

Because this manuscript is specifically focused towards reporting whether the effects found earlier in laboratory studies were replicated by parallel effects collected during this study’s real-world living conditions, we are presenting only the *n*-back and Go/No-Go assessments herein [5,12]. Furthermore, the other study tasks represent very different cognitive processes, which are more exploratory in nature. The other tasks will be reported in a future manuscript and will reference this publication as a related paper from the same clinical trial.

For the acute results, we specifically gathered data on the pre- and post-administration *n*-back and Go/No-Go tasks at the start of the study. Thereafter and throughout the entire study duration, daily compliance checks were administered via the participant’s smartphone using Metricwire (Version 4.9.9, Kitchener, ON, Canada) [31].

### 2.2. Participants

**Figure 1:**
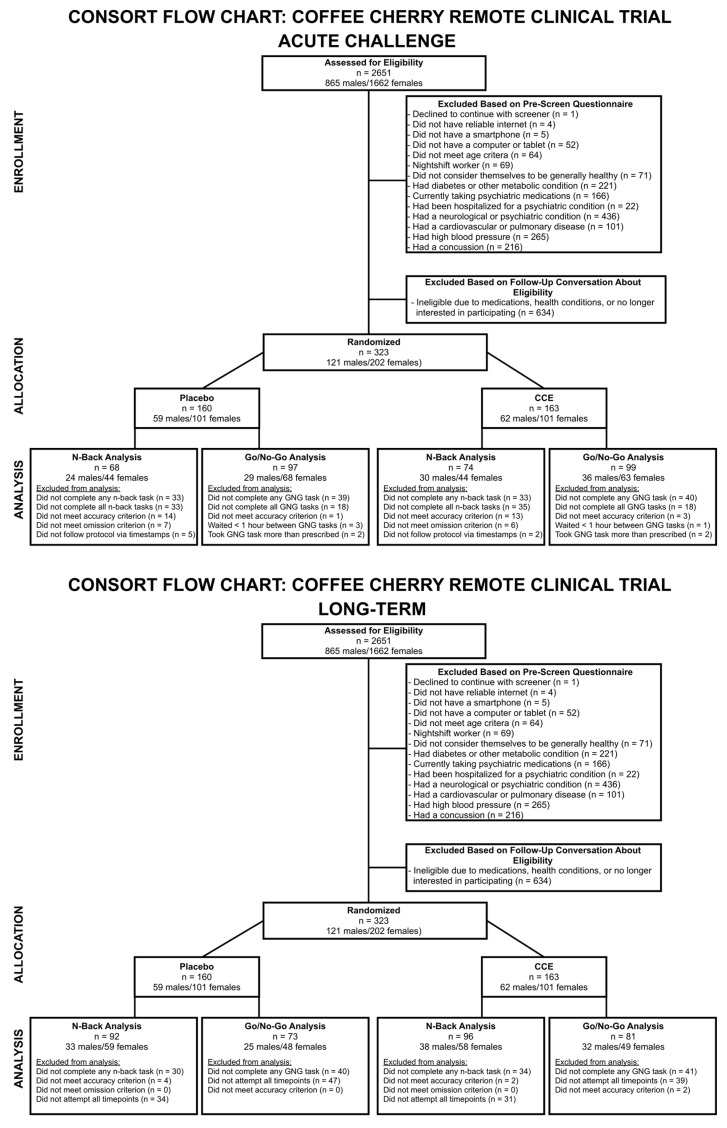
CONSORT flow chart for the remote coffee cherry clinical trial. Participants that engaged in the acute challenge are the same participants enrolled in the longitudinal study (i.e., they are not a separate pool of participants, but some participants were excluded for certain analyses based on inclusion/exclusion criteria).

Participants were recruited through flyers and social media posts. Interested participants took an online screener through Qualtrics. Power analyses using previously reported effect sizes [5] indicated that we would need *N* = 153. To be eligible, participants had to reside in the United States, be between 40 and 65 years of age, with no known psychiatric or neurological conditions, and be generally healthy. Additionally, they had to have a smart phone and a computer or compatible tablet to perform the cognitive tasks required as part of the study along with self-reported computer literacy. Participants were excluded if they were taking medications known to alter cognitive functioning (i.e., psychotropic medications such as fluoxetine, benzodiazepines), had a metabolic condition such as diabetes or hypo/hyperthyroidism, were a nightshift worker, had any food allergies, or had other medical conditions that may impact their ability to perform the study or metabolize the study material. Participants who met inclusion criteria were contacted via email and scheduled for a video call to explain the study and obtain informed consent. Participants were sent the informed consent form in advance of the call to review. Informed consent consisted of a detailed explanation of the study as well as reviewing the informed consent document with the participant via video conference. Participants digitally signed the informed consent document. After consenting, participants were sent study materials which included a schedule of study dates, the study material, and a magnet reminder (to be placed on their refrigerator). In addition, participants were required to sign up with a momentary assessment software, MetricWire (Version 4.9.9, Ontario, Canada) [31], which provided daily reminders and surveys to help ensure compliance to the study protocol. Participants were also sent detailed instructions to download and install the cognitive challenge testing platform, Inquisit Player [16], along with two sample tests to ensure compatibility with their device. Participants were instructed to complete the cognitive challenges prior to taking their study materials, and to not eat or drink anything other than water prior to completing the challenges. Participants were compensated commensurate with their participation ($10 for completion of the first assessment, $15 for completion of the second assessment, $20 for completion of the third assessment, $25 for completion of the fourth assessment, $30 for completion of the fifth assessment, and $25 for completion of >75% of the daily compliance checks), with a maximum payout of $125. Compensation was paid at the end of the study, or after two consecutive missed assessments. For enrollment and allocation, please see Figure 1. Participants were enrolled between February 2022 and October 2022. The trial concluded because recruitment goals were achieved.

Participant randomization was performed by study team members (MK, JJ, JR, CW), using stratified permuted block randomization with the R package ‘blockrand’ to ensure that participants were equally represented in treatment groups based on their sex assigned at birth and implemented in a randomization spreadsheet. Group assignment (i.e., group A or group B) was not revealed until a participant’s study specific identification number was entered into a spreadsheet on the day that their study materials were scheduled to ship. Randomization code was implemented by MK. Unblinding occurred after study analyses were completed (May 2023). All participants and study team members were blind to which intervention they were assigned to.

### 2.3. Tasks

#### 2.3.1. N-Back

A Millisecond Software, LLC. (Version 6.5.1; Seattle, WA, USA) [16] custom script was generated for the *n*-back task. Three conditions were randomized within this task: 0-, 1-, and 2-back. During the 0-back condition, participants were shown a sequence of stimuli (i.e., geometric shapes such as a triangle or circle), and were asked to indicate whether or not the currently presented stimulus matched a pre-defined “target” shape. Target shapes were randomized, and all targets were white outlined shapes presented on a black background. Because the mental processes involved in performing the 0-back condition are decidedly different than those required during the 1- and 2-back conditions, the 0-back data collected in this study were only used as a means of establishing each participant’s minimum qualification for inclusion in the overall *n*-back assessment. Therefore, 1- and 2-back data from participants whose 0-back data failed to fulfill our predetermined minimum 0-back performance were excluded from our final analyses (see details in Results section below). The “1-back” assessment required the participants to indicate whether or not each currently presented stimulus was the same shape as the one that immediately preceded it, and the “2-back” assessment required the participants to indicate whether or not each currently presented stimulus was the same shape as the one that had been presented two trials before. Difficulty increased from 1- to 2-back conditions. After participants read the instructional screens, they received one block of 10 trials of practice for each of the respective ‘n’ level tests prior to initiating the actual assessment. Once a practice was completed, participants had the option to repeat the practice block. All practice blocks consisted of 10 trials with a 3:7 target:non-target ratio. After completing all practice blocks, participants received two test blocks per level of ‘*n*’. Testing blocks were randomized. Each trial presented the shape for 500 ms, followed by a 2500-ms delay before presenting the next shape. Participants had the full 3000 ms to respond. Participants were asked to respond to every trial by pressing the number ‘1’ if the stimulus was a target, or the number ‘4’ if it was not a target. Each testing block consisted of 30 trials with a 9:21 target:non-target ratio. Target trials were randomized within all blocks. By way of definition, target trials were defined as trials in which the presented stimulus matched the stimulus specified by each respective *n*-back condition, and non-target trials were those in which the presented stimulus did not match. Correct responses were defined as those that were accurately categorized (i.e., those instances when a target was correctly selected as a target and those instances when a non-target was correctly determined to be a non-target). Here, we calculated total accuracy as well as accuracy within each trial type (i.e., target and non-target). A false alarm was defined as incorrectly identifying a non-target as a target stimulus. In instances when a participant failed to respond at all to a stimulus within the allocated 3000 ms, their lack of response was recorded as an ‘omission’.

#### 2.3.2. Go/No-Go

A Millisecond Software, LLC. (Version 6.5.1; Seattle, WA, USA) custom script was generated for the Go/No-Go task. Participants were asked to press the ‘spacebar’ for stimuli that changed from trial to trial (e.g., from ‘x’ to ‘y’ or from ‘y’ to ‘x’; considered a “Go” trial) and were asked to refrain from responding for stimuli that stayed the same (e.g., from ‘x’ to ‘x’ or from ‘y’ to ‘y’; considered a “No-Go” trial). Participants were introduced to the task via instructional screens. They then performed a practice block of trials during which there were 12 ‘Go’ trials and six ‘No-Go’ trials for a total of 18 trials. Participants were allowed to take the practice block as many times as they wanted. Following the practice block, there were four test blocks consisting of 60 stimuli (one starting stimuli, 53 ‘Go’ trials, and six ‘No-Go’ trials). ‘No-Go’ trials were always randomized. Stimuli were presented for 500 ms followed by a 500-ms blank screen and a 500-ms intertrial interval. A 15-s rest interval was provided between blocks. By way of definition, a ‘hit’ was a correct response to a ‘Go’ trial (i.e., the participant pressed the ‘spacebar’). The ‘hit rate’ was the number of hits divided by the total number of trials. An ‘omission’ was defined as the number of times the participant did not press the ‘spacebar’ for a ‘Go’ trial, and a ‘commission’ was defined as the number of times the participant incorrectly pressed the ‘spacebar’ for a ‘No-Go’ trial.

### 2.4. Data Analysis

Omnibus linear mixed effects (LME) modeling with autoregressive covariance structure was implemented to analyze the data. Main effects of time and treatment were assessed, as well as time-by-treatment interactions. A treatment-by-time interaction generally means that the treatment had differential effects between the groups over time. LME was chosen because it is a robust statistical method designed for use with multi-timepoint data. Post-hoc pairwise comparisons with Bonferroni correction were implemented to determine significant differences between placebo and CCE groups when necessary for the acute analyses. Post-hoc univariate ANOVAs were implemented on significant longitudinal effects to characterize and parse out the nature of the effect(s).

## 3. Results

### 3.1. Acute Challenge Results

#### 3.1.1. N-Back

**Figure 2:**
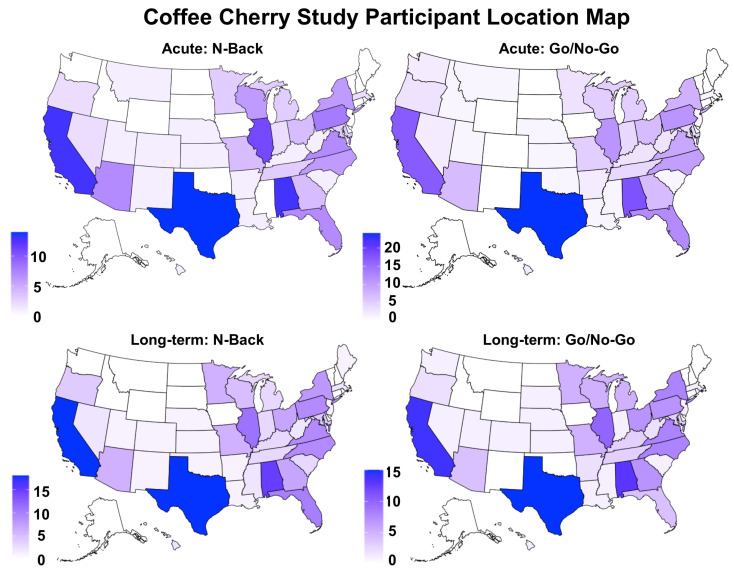
Depiction of participant location for the *n*-back and Go/No-Go tasks.

Data were excluded if participants (a). did not adhere to the study protocol as assessed by submission timestamps; (b). did not complete both the pre- and post-administration *n*-back tasks; (c). did not achieve at least 50% accuracy across all trials (i.e., 90 out of 180 successful responses); or (d). had more than 15% omissions (i.e., nine trials) during the 0-back control condition. Because the *N*-back task was administered twice at each of the study timepoints (i.e., as the first and also as the last task of the entire cognitive challenge battery), we had visibility into the exact time elapsed between the end of the first cognitive challenge (i.e., the second *n*-back of the pre-administration) and the beginning of the second cognitive challenge (i.e., the first *n*-back of the post-administration). As such, we only included those participants whose elapsed times between the end of the first cognitive challenge and the beginning of the second cognitive challenge were between 45 min and 3 h (M ± SD: M_placebo_ = 1.284 ± 0.232, M_cce_ = 1.324 ± 0.309; F (1, 140) = 0.755, *p* = 0.387). The final sample size for the *N*-back analysis was *N* = 142 (*n*_placebo_ = 68, *n*_cce_ = 74). Groups did not differ on race, sex assigned at birth, education, employment type, marital status, household size, household income, geographical region (see Figure 2), or age (see Table 1 for descriptive demographic statistics).

**Figure 3:**
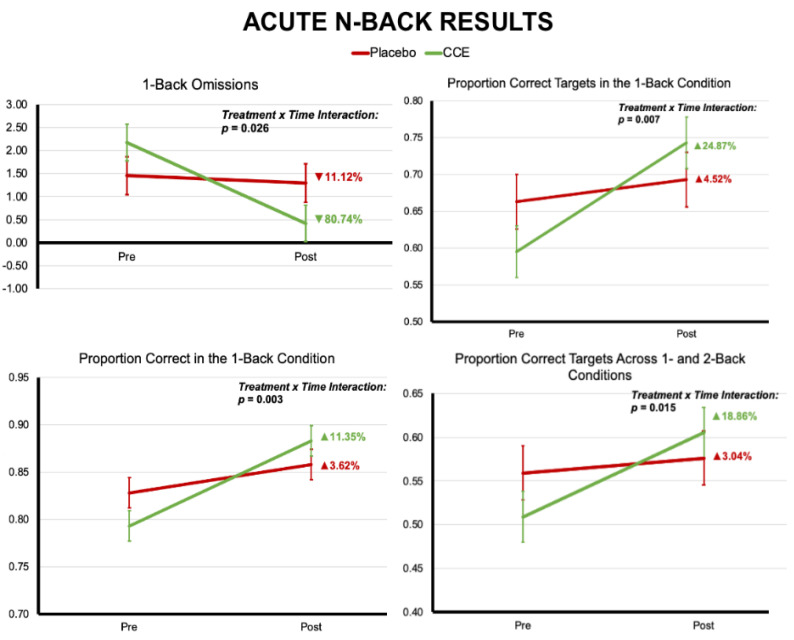
Results from the acute *n*-back challenge.

Results indicate that at 1-h post-ingestion a single dose of CCE significantly reduced the number of omissions for the 1-back trials as compared to placebo (F (1, 140) = 5.096, *p* = 0.026). CCE also showed significant improvement compared to placebo in accuracy for the 1-back trials when examining correct trials of any type (i.e., target and non-target; F (1, 140) = 9.446, *p* = 0.003). CCE also showed significant improvement compared to placebo in accuracy for the 1-back trials when examining only the correct target trials (F (1, 140) = 7.471, *p* = 0.007) (see Figure 3 and Table 2 for all statistical results and outcome measures). Descriptively, the groups differed greatly on number of omissions during the *n*-back, such that the CCE group reduced their number of omissions by 80.7% while the placebo group decreased omissions by 11.1%. Additionally, when examining all 1-back trials, the CCE group improved accuracy by 11.3% while the placebo group improved by 3.6%. When examining only the correct target trials during this condition, the CCE group improved by 24.9% compared to 4.5% for the placebo group. It is worthy of note that the CCE group had significantly better baseline performance than the placebo group during our acute assessments. Consequently, it would be reasonable to expect that the placebo group would have been better positioned for improvement (i.e., greater improvements in accuracy and/or less omissions) over time. However, despite these initial disadvantages, the CCE group significantly outperformed the placebo group.

The control condition (i.e., 0-back), as defined, is primarily a task of perception [32,33] that involves the use of mental processes that are distinctly different from those employed during 1-back. It should be noted that the 0-back was incorporated into this study as an *n*-back training aid and as a qualifier to determine whether to include or not include participants’ data in our statistical evaluations of *n*-back. During the 0-back, there was a significant treatment-by-time interaction such that the placebo group showed marginal increases in accuracy (0.9%) while the CCE group had marginal decreases (−2.2%; F (1, 140) = 4.109, *p* = 0.045). These latter results are likely not clinically significant, given the low magnitude of the effect, but are more likely driven by directionally opposite effects (i.e., increase versus decrease), which seems to be due to fewer false alarms in the placebo group post-administration (a 40.7% decrease compared to a 10% increase in the CCE group).

Groups did not differ on any acute accuracy measurements during the 2-back condition. However, when combining 1- and 2-back conditions, there was a trending interaction effect whereby across all trial types (i.e., non-target and target), CCE improved by 8.8% while placebo improved by 5% (F (1, 140) = 3.388, *p* = 0.068). Notably, there was a significant treatment-by-time interaction for the proportion of correct target trials in which CCE demonstrated over six times the improvement compared to placebo (18.9% improvement compared to 3.0%; F (1, 140) = 6.032, *p* = 0.015). There were no significant differences for any non-target trials, regardless of condition. Furthermore, although we observed reaction time improvements versus baseline for the CCE group, there were no significant effects for reaction time, other than significant main effects of time.

#### 3.1.2. Go/No-Go

Participants were excluded if (a.) they did not adhere to the study protocol as assessed by submission times (i.e., participants were excluded if their submission times were under 1.5 h; M ± SD: M_placebo_ = 2.34 ± 0.84; M_cce_ = 2.30 ± 0.89; F (1, 194) = 0.98, *p* = 0.755); (b.) they did not complete both the pre- and post-administration go/no-go tasks; or (c.) they did not achieve at least 25% accuracy across all trials. The final sample size for the go/no-go analyses was *N* = 196 (*n*_placebo_ = 97, *n*_cce_ = 99). Groups did not differ on race, sex assigned at birth, education, employment, marital status, household size, household income, or geographical region, but did differ slightly on age (M ± SD: M/_placebo_ = 49.46 ± 7.15, M_cce_ = 51.88 ± 7.57; F (1, 193) = 5.238, *p* = 0.023) (Table 1).

**Figure 4:**
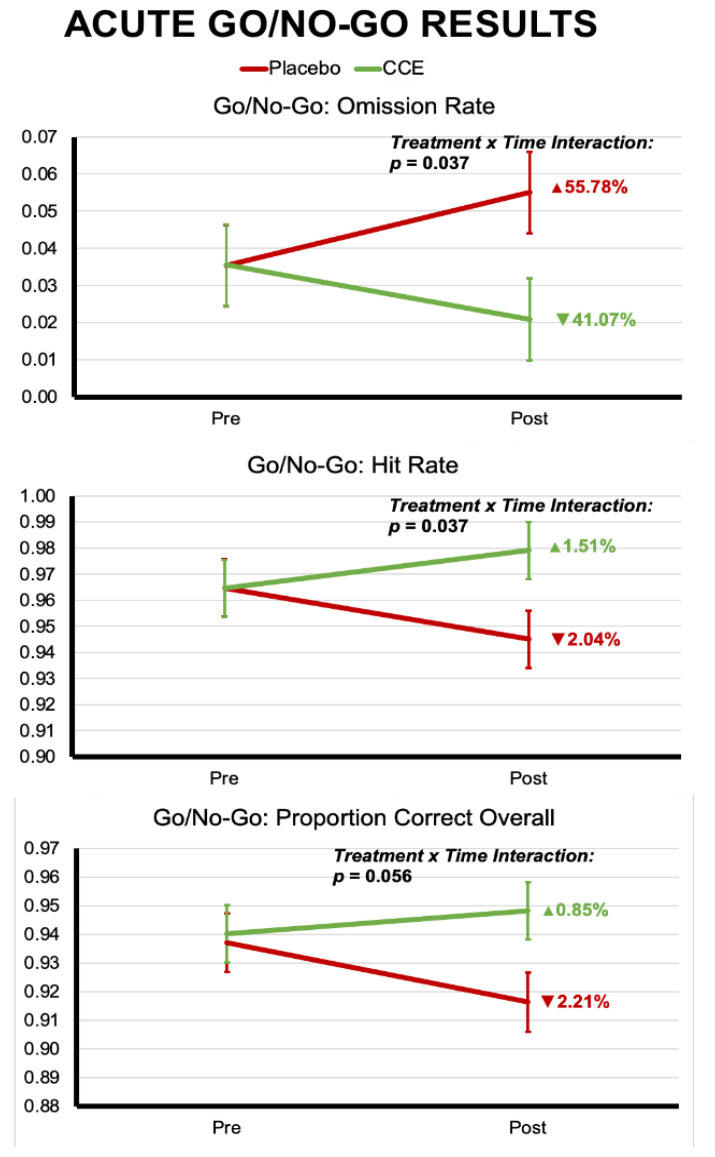
Acute results from the Go/No-Go task.

LME modeling showed that the CCE group had a significantly decreased go/no-go omission rate of −41.1% compared to an increased omission rate of 55.8% for the placebo group (F (1, 194) = 4.417, *p* = 0.037). Importantly, go/no-go omissions can be indicative of accrual of stress, fatigue, loss of focus, attention, concentration, motivation, and/or accuracy. Indeed, due to the various inhibitory mental processes being measured, changes in the number of mistakes (meaning omissions and commissions) arguably provide the most important information related to cognitive performance during the go/no-go assessment. We also observed a significant treatment-by-time interaction for hit rate (correct responses), whereby the CCE group improved 1.5% while the placebo group declined 2.0% (F (1, 194)= 4.417, *p* = 0.037) (Figure 4). LME modeling also revealed strong trend-level significance (F (1, 194) = 3.699, *p* = 0.056) for a treatment-by-time interaction, such that CCE participants improved overall proportion correct performance by 1% while placebo participants decreased by 2%. There were no significant interaction effects for commission rate, or any of the reaction time analyses. However, for both the commission rate and for all reaction time analyses, there was a significant effect of time, such that both groups had faster reaction times, but had higher commission rates (Table 3).

### 3.2. Longitudinal (Long-Term) Challenge Results

#### 3.2.1. N-Back

Participants were excluded if they did not attempt to complete all five timepoints in the study. Timepoints that had <50% accuracy across all trials (i.e., <90 out of 180 successful responses) or >15% omissions (i.e., nine trials) during the 0-back control condition were also excluded. The final sample size for the *n*-back analysis was *N* = 188 (*n*_placebo_ = 92, *n*_cce_ = 96). Groups did not differ on race, sex assigned at birth, education, employment type, marital status, household size, household income, geographical region (see Figure 2), or age (see Table 4 for descriptive demographic statistics).

**Figure 5:**
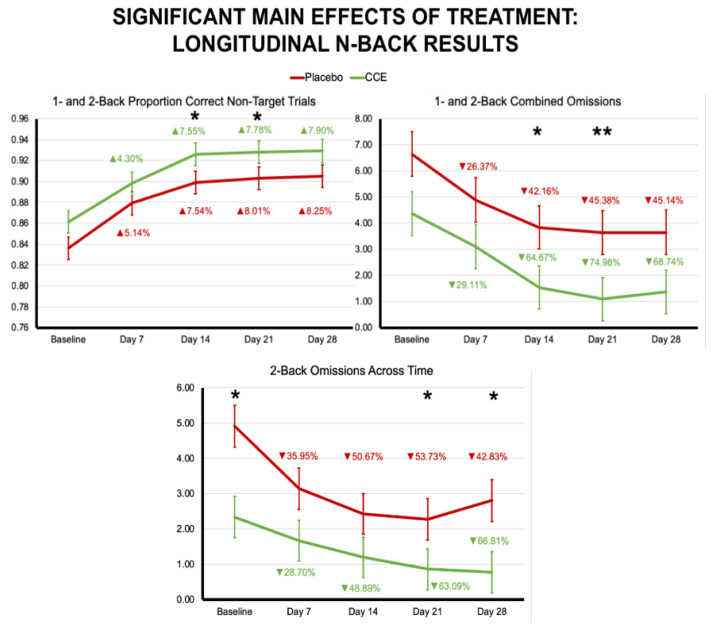
Main effects of treatment from the longitudinal analysis of the *n*-back task. Percentages indicated are calculated from baseline. Asterisks indicate level of significance from follow-up univariate ANOVAs at each timepoint (* = *p* < 0.05; ** = *p* < 0.01 ).

Results indicate that there was a significant effect of treatment for the proportion of correct non-target trials and number of omissions across all trials, in addition to the proportion of correct non-target trials and number of omissions in the 2-back condition (please see Table 5 for statistical reporting, Figure 5). It should be noted that there were baseline differences between groups for the 2-back non-target correct trials and the 2-back omissions. Descriptively, the CCE group had significantly better performance at baseline. Again, it would be reasonable to expect that over time, the placebo group would have been better positioned for improvement (i.e., greater improvements in accuracy and/or less omissions). However, this was not the case. For the 2-back omissions, the follow-up univariate ANOVAs suggest that at Day 21 and Day 28, the CCE group continued to have less omissions (i.e., less mistakes leading to better performance) than the placebo group. When examining both the omissions and the correct non-target trials for combined 1- and 2-back conditions, the CCE group outperformed placebo on Days 14 and 21 for both. Additionally, there was a trend toward a time-by-treatment interaction for the combined 1- and 2-back proportion correct target trials, such that CCE improved 14.7%, 24.9%, 25.7%, and 28.9% from baseline on Days 7, 14, 21, and 28, respectively, whereby placebo only improved 6.5%, 7.8%, 13.0%, and 13.0% (F (1, 665.183) = 2.235, *p* = 0.064).

**Figure 6:**
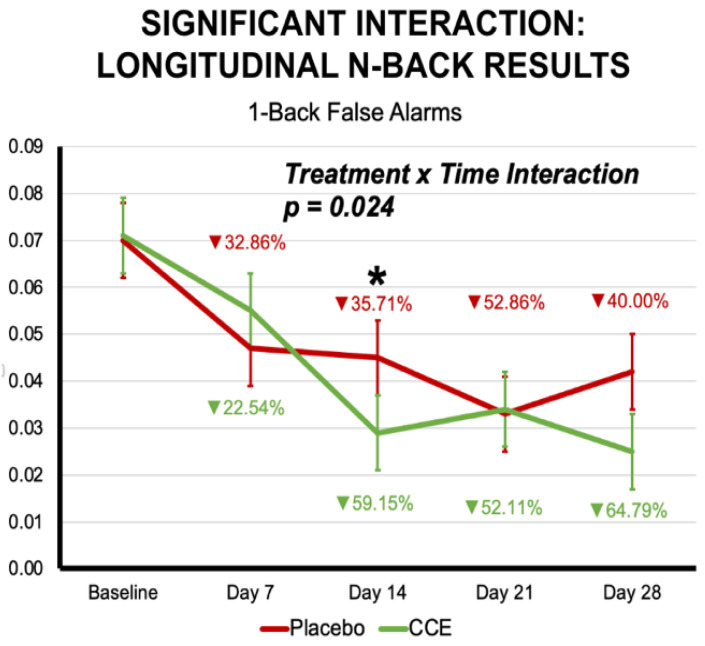
Interaction effect from the longitudinal analysis of the *n*-back task suggesting that CCE and placebo groups demonstrated different trajectories over time. Specifically, CCE showed a more pronounced decrease in false alarms at Day 14 and showed decreases from Day 21 to Day 28, whereas placebo did not. Percentages indicated are calculated from baseline. Asterisks indicate level of significance from follow-up univariate ANOVAs at each timepoint (* = *p* < 0.05 ).

Finally, there was a significant time-by-treatment interaction for the number of false-alarms during the 1-back condition (Figure 6). Descriptively, the CCE group decreased the number of false alarms (i.e., less mistakes which would lead to better performance) by 22.5%, 59.2%, 52.1%, and 64.8%, respectively. In comparison, the placebo group decreased the number of false alarms by 32.9% on Day 7 compared to baseline, 35.7% on Day 14, 52.9% on Day 21, and 40.0% on Day 28. Follow-up univariate ANOVAs indicated that at Day 14, the CCE group had significantly fewer false alarms compared to the placebo group (i.e., a decrease of 59.2% for CCE from baseline compared to 35.7% for placebo). There was also a trend-level significant interaction for both the number of omissions during the 0-back condition (*p* = 0.088) and proportion correct for all 1-back trials (*p* = 0.078). Together, in addition to what we observed acutely, these data suggest that the CCE group may have significant longitudinal improvements related to accuracy compared to the placebo group.

#### 3.2.2. Go/No-Go

Participants were excluded if they did not complete any Go/No-Go task or did not attempt the Go/No-Go task at every timepoint. Timepoints that did not meet the accuracy criterion (>25% accuracy across all trials) were excluded. The final sample size for the go/no-go analyses was *N* = 154 (*n*_placebo_ = 73, *n*_cce_ = 81). Groups did not differ on race, sex assigned at birth, education, employment, marital status, household size, household income, or geographical region, but did differ slightly on age (M ± SD: M_placebo_ = 48.36 ± 6.82, M_cce_ = 51.09 ± 7.92; F (1, 151) = 5.182, *p* = 0.024) (Table 4).

Results indicate that there were no significant main effects of treatment, nor were there any significant treatment-by-time interactions (Table 6). There were significant effects of time for mean overall reaction time and mean hit reaction time, such that both groups improved their reaction time over the course of the 28 days.

## 4. Discussion

Here, we present a novel remote clinical trial in which we demonstrate results that are concordant with previous, laboratory-based studies, while also extending the current literature. This clinical trial utilized a very diverse, generally healthy study population aged 40–65 years old that participated remotely and electronically from across the United States. As such, we expected more noise in the data, and considered whether we might observe smaller effect sizes compared to the similar studies previously conducted in a clinical setting due to the impacts of the ‘real-world’ testing environment that, by definition, would be less rigorously controlled and less consistent across subjects. As expected, the generated data was indeed somewhat noisier than what would be anticipated from a study conducted in a typical laboratory setting. In retrospect, and despite the larger *N*, taking all of the unexpected variables into consideration, this study demonstrated strong statistical trends that would likely have yielded even stronger results with additional participants. The outcomes nonetheless add significantly to the body of research related to the acute impacts of CCE.

Our current acute data support greater improvements in accuracy during the 1-back condition of the *n*-back task. Also, we noted significant reductions in omissions when completing the acute 1-back task, which led to greater improvements in accuracy for the CCE group compared to the placebo group. This notable reduction in omissions may suggest the CCE participants avoided mentally “locking up” when faced with repeated, time-pressured response challenges. Furthermore, we demonstrated that whole coffee cherry extract showed greater reductions in omissions, and greater improvements in accuracy for the Go/No-Go task with regard to mean hit rate and a strong trend toward outperforming placebo in overall accuracy after a single-dose acute administration [5]. These results are in alignment with our hypotheses that CCE would outperform placebo, in alignment with previous laboratory-based investigations.

We did note that the placebo group outperformed the CCE group acutely during the 0-back task. This may reflect psychological process differences, as the 0-back task is essentially a perceptual task focused on identifying a single item across a host of trials, and not a cognitive assessment. There was no difference in the longitudinal data. Comparatively, the 1- and 2-back conditions were true cognitive challenges, requiring participants to keep a running mental log (memory) of stimuli in order to perform the task correctly. Furthermore, the differences observed during the acute challenge 0-back condition are likely due to directional differences, and do not appear to reflect an effect of magnitude.

We did not find any significant interaction effects with regard to reaction time on any of the measures (i.e., in this case, both groups improved their reaction time), although, as reported earlier, we did observe reaction time improvements in the CCE group when compared to baseline. This is in contrast to statistically significant reaction time decreases that were reported in earlier non-acute studies longitudinally [5,12]. We are not entirely surprised by how the data presents here, given the different types of devices used to complete the task, coupled with the larger-than-average reaction time improvements observed in the placebo group compared to those observed in prior studies and, in particular, given the clear signal we saw acutely on improvements in accuracy for the CCE group. It is important to note that, unlike our generally healthy current study population, previous laboratory-based studies had older populations with reported cognitive decline. The current study extends the literature by using a younger population (ages 40–65) that was generally healthy without any cognitive impairments. Furthermore, the diversity of the current sample along with the design of the study is much more representative and inclusive than previous studies, increasing the generalizability of the results.

**Figure 7:**
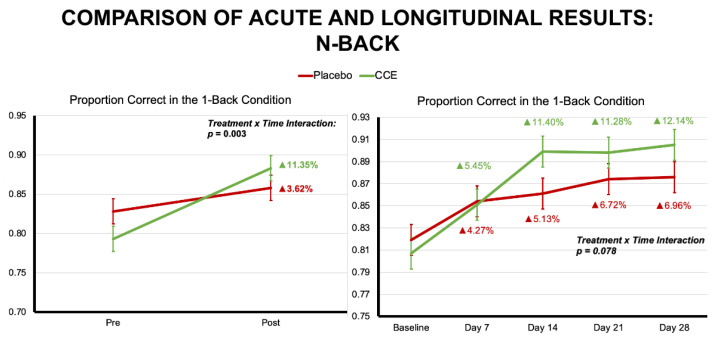
Comparison of the acute and longitudinal results for the 1-back condition for overall proportion correct.

Our longitudinal results suggest that the CCE group decreased false alarms during the 1-back task, with significant differences emerging by Day 14. As mentioned, the acute 1-back proportion correct across all trials demonstrated a significant interaction, such that the CCE group outperformed placebo. Longitudinally, we saw a similar pattern that approached significance (Figure 7). Together, this provides additional important evidence of CCE’s acute and longer-term efficacy related to cognition, especially given that this study was conducted outside of strict laboratory conditions that would be expected to potentially yield higher levels of effort, concentration, and focus that are not necessarily typical of the real world.

Despite the lack of interaction effects for the 2-back condition, both acutely and longitudinally, we did observe some notable main effects of treatment. Specifically, the 2-back proportion of correct non-target trials and the number of omissions demonstrated main effects of treatment. While follow-up univariate ANOVAs directed our attention toward baseline differences for both results, CCE had fewer longitudinal omissions at Day 21 and Day 28 compared to placebo. These results were similar to the main effects of treatment that were demonstrated for proportion of correct non-target trials across all conditions and also for the number of omissions across all conditions. However, in the case of omissions and proportion correct of non-target trials across all conditions, there were no baseline differences, suggesting that CCE had greater accuracy at Day 21 for both metrics, and additionally at Day 14 for omissions. Together, these data suggest that the CCE group had greater accuracy during the 2-back condition (and across all conditions) with regard to correct non-target trials as well as omissions.

These data provide replication and increase our understanding of the effects of CCE on working memory. It is important to emphasize that, other than the acute data gathered on Day 1 as reported earlier herein, the remainder of this study was focused upon measuring cumulative effects. This is why subjects were instructed to refrain from taking any study materials before engaging in their assessments throughout the balance of the study.

**Figure 8:**
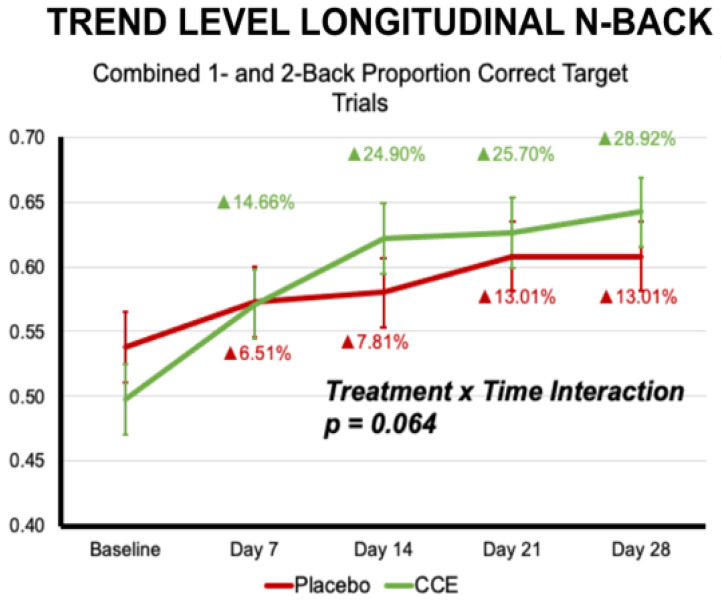
Trend-level interaction effect from the longitudinal analysis of the *n*-back task suggesting that CCE and placebo groups demonstrated different trajectories over time. Specifically, CCE showed a more pronounced increase in proportion correct during the first two weeks and continued to improve, whereas placebo did not. Percentages indicated are calculated from baseline.

Several observations are worthy of note. First, we determined our estimated sample size a priori based on previous effect sizes that were determined in laboratory studies that were controlled very differently from the ‘real-world’ set-up we executed here. Based upon several of the observed strong longitudinal trends that approached significance (Figure 8), a larger sample size might be necessary to detect the effect. We will take that into account when designing future protocols. Second, we may have underestimated the amount of “noise” we would encounter in the data. As partially anticipated, our remotely administered assessment data did have substantially more noise than earlier clinical settings due to numerous extraneous variables that are attendant to the “real world” and are absent in a laboratory clinical setting. In this study, subjects (a diverse group from across the United States) performed their assessments in their own homes, on their own equipment, and under different conditions. As such, future longer-term studies should consider these study protocol differences when pursuing a remote clinical trial. This complication was not as significant in the acute measures. However, the data became noisier over the 4-week longitudinal timeframe due, at least in part, to the many variables introduced by distanced, non-laboratory assessments, not to mention various technical challenges, web-enabled software glitches, and user errors. These challenges are inherent in a remote clinical trial (as they are inherent in real life), and do not necessarily represent weaknesses in the approach, but are, by their very nature, strengths that yield clinical data outcomes that are far more likely to align to a person’s real-life experience outside of the lab. The benefits (i.e., accessibility, increased generalizability, more diverse samples) are significant and should not be underestimated. Indeed, we perceive them as potentially necessary for the advancement of nutraceutical research.

Future investigation is needed to better understand the physiological mechanisms underlying the observed improvements in cognitive function. For example, studies have demonstrated the potential for coffee-cherry-derived products to alleviate brain inflammation in mice through inflammatory signaling pathways and the gut–brain axis [10]. Other research has demonstrated neurophysiological changes through the use of neuroimaging [5]. Still other research has suggested a role for brain-derived neurotrophic factor [4,34]. Comprehensive, multi-modal studies are necessary to better understand the neurophysiological mechanisms that may support improvements in brain function.

## 5. Conclusions

We believe that this current study is a contributory step towards transforming the clinical trial landscape, especially for brain health supplements. The world is full of supplements with positive outcomes in laboratory settings that do not fulfill their promises when taken in the messiness of real life. Future studies should expand on this line of inquiry by conducting adequately powered, accessible remote clinical trials that take into consideration, and recognize as beneficial, the difficulties and extraneous variables that are specific to conducting such trials that mirror much more closely real-life circumstances. Demonstrating real-world results that are consistent with, and expand, results obtained from previous laboratory-based studies provides more confidence that the effects of CCE on focus, attention, concentration, and accuracy are not merely a product of a sterile, artificial environment, but are in fact real effects that can be experienced at home.

Finally, we feel that these data serve to enrich the growing body of evidence that CCE may support and potentially enhance brain health and function in older adults. Given the burgeoning global interest in brain health supplementation, it is critical that more robust research efforts are devoted toward characterizing the nature and efficacy of these supplements. This is especially true given that many brain-health studies that have been conducted were observational, underpowered, and/or not generalizable because of the laboratory setting [35]. A recent commentary has touched on the importance of conducting both rigorous laboratory studies as well as longer-term observational studies that increase generalizability and leverage tools that allow for improved assessment of study coherence [35,36,37,38]. Thus, our study provides an attempt to bridge this gap by using methodologies previously applied in controlled laboratory settings but now implemented in real-world situations to provide generalizable data.

## Figures and Tables

**Table 1:** Demographic information and statistics for participants included in both the *n*-back and Go/No-Go analyses for the acute challenge.

	*N*-Back				Go/No-Go			
	Placebo (*n* = 68)	CCE (*n* = 74)	Statistic	*p*	Placebo (*n* = 97)	CCE (*n* = 99)	Statistic	*p*
Ethnicity								
American Indian or Alaskan Native, Hispanic or Latino, Asian or Asian American	2.94%	2.70%	χ^2^ = 4.072	0.771	3.09%	4.04%	χ^2^ = 7.381	0.598
Asian or Asian American	2.94%	2.70%			5.15%	3.03%		
Black of African American	4.41%	6.76%			2.06%	4.04%		
Hawaiian or Pacific Islander, White	0.00%	1.35%			0.00%	2.02%		
Hispanic or Latino	5.88%	9.46%			5.15%	6.06%		
White	82.35%	75.68%			81.44%	79.80%		
Mixed race	0.00%	1.35%			2.06%	1.01%		
Preferred not to say	1.47%	0.00%			1.03%	0.00%		
Education								
High School Diploma	4.41%	2.70%	χ^2^ = 2.097	0.836	3.09%	2.02%	χ^2^ = 2.783	0.947
Post-secondary Non-Degree Award	0.00%	0.00%			1.03%	1.01%		
Associate’s Degree	5.88%	9.46%			7.22%	11.11%		
Some College, No Degree	19.12%	21.62%			19.59%	18.18%		
Bachelor’s Degree	36.76%	37.84%			35.05%	34.34%		
Master’s Degree	23.53%	22.97%			22.68%	24.24%		
Doctoral or Professional Degree	10.29%	5.41%			9.28%	6.06%		
Other	0.00%	0.00%			0.00%	1.01%		
No Response	0.00%	0.00%			2.06%	2.02%		
Employment								
Homemaker	10.29%	4.05%	χ^2^ = 8.806	0.551	9.28%	3.03%	χ^2^ = 11.732	0.303
Student	0.00%	1.35%			0.00%	1.01%		
Employed for Wages	63.24%	62.16%			62.89%	65.66%		
Military	0.00%	1.35%			0.00%	1.01%		
Other	0.00%	1.35%			0.00%	1.01%		
Out of Work—Looking	5.88%	4.05%			4.12%	3.03%		
Out of Work—Not Looking	0.00%	4.05%			0.00%	4.04%		
Retired	10.29%	9.46%			8.25%	9.09%		
Self-Employed	7.35%	6.76%			10.31%	8.08%		
Unable to Work	2.94%	4.05%			3.09%	1.01%		
No Response	0.00%	1.35%			2.06%	3.03%		
Marital Status								
Divorced	11.76%	16.22%	χ^2^ = 7.477	0.279	10.31%	17.17%	χ^2^ = 7.459	0.281
Married or Domestic Partnership	72.06%	56.76%			68.04%	56.57%		
Other	1.47%	0.00%			1.03%	0.00%		
Separated	1.47%	2.70%			4.12%	2.02%		
Single, Never Married	11.76%	14.86%			13.40%	16.16%		
Widowed	1.47%	8.11%			1.03%	5.05%		
No Response	0.00%	1.35%			2.06%	3.03%		
Household Size								
1 Person	16.18%	25.68%	χ^2^ = 7.130	0.203	13.40%	23.23%	χ^2^ = 9.394	0.153
2 People	35.29%	33.78%			35.05%	33.33%		
3 People	14.71%	16.22%			17.53%	21.21%		
4 People	20.59%	8.11%			18.56%	6.06%		
5 People	7.35%	10.81%			8.25%	8.08%		
6 or More People	5.88%	4.05%			5.15%	5.05%		
No Response	0.00%	1.35%			2.06%	3.03%		
Household Income								
Less than $25,000	4.41%	8.11%	χ^2^ = 9.195	0.420	2.06%	5.05%	χ^2^ = 10.928	0.281
$25,000–$34,999	2.94%	5.41%			3.09%	6.06%		
$35,000–$49,999	5.88%	9.46%			7.22%	9.09%		
$50,000–$74,999	10.29%	14.86%			14.43%	16.16%		
$75,000–$99,999	25.00%	22.97%			22.68%	28.28%		
$100,000–$149,999	27.94%	20.27%			28.87%	18.18%		
$150,000–$199,999	7.35%	12.16%			7.22%	10.10%		
$200,000 or more	10.29%	2.70%			9.28%	2.02%		
I prefer not to say	5.88%	2.70%			3.09%	2.02%		
No Response	0.00%	1.35%			2.06%	3.03%		
Geographical Region								
Midwest—IA, IL, IN, KS, MI, MN, MO, ND, NE, OH, SD, WI	23.53%	25.68%	χ^2^ = 4.221	0.518	23.71%	19.19%	χ^2^ = 0.904	0.970
Northeast—CT, DC, DE, MA, MD, ME, NH, NJ, NY, PA, RI, VT	16.18%	10.81%			12.37%	13.13%		
Southeast—AL, AR, FL, GA, KY, LA, MS, NC, SC, TN, VA, WV	27.94%	33.78%			34.02%	36.36%		
Southwest—AZ, NM, OK, TX	13.24%	17.57%			14.43%	16.16%		
West—AK, CA, CO, HI, ID, MT, NV, OR, UT, WA, WY	19.12%	10.81%			13.40%	12.12%		
No Response	0.00%	1.35%			2.06%	3.03%		
Sex Assigned at Birth								
Male	24	30	χ^2^ = 0.414	0.520	29	36	χ^2^ = 0.924	0.336
Female	44	44			68	63		
Age (M ± SD)	50.26 ± 7.49	51.24 ± 7.47	F (1, 140) = 0.606	0.438	49.46 ± 7.15	51.88 ± 7.57	F (1, 193) = 5.238 *	0.023

* = One participant declined to provide their specific age, and only responded to an age range question.

**Table 2:** Results of linear effects modeling for all dependent outcome measures for the acute challenge *n*-back task.

	Pre-Adminstration				Post-Administration				Effect of Treatment		Effect of Time		Treatment Time	
	Placebo (*n* = 68)		CCE (*n* = 74)		Placebo		CCE							
	M	SE	M	SE	M	SE	M	SE	F (1, 140)	*p*	F (1, 140)	*p*	F (1, 140)	*p*
Accuracy														
Proportion Correct *N* = 1 & *N* = 2 Combined	0.765	0.014	0.747	0.013	0.803	0.014	0.813	0.013	0.049	0.825	48.030	<0.001	3.388	0.068
Proportion Correct *N* = 1 & *N* = 2 Target Trials	0.559	0.031	0.509	0.029	0.576	0.031	0.605	0.029	0.068	0.795	12.201	<0.001	6.032	0.015
Proportion Correct *N* = 1 & *N* = 2 Non-Target Trials	0.853	0.013	0.849	0.013	0.901	0.013	0.902	0.013	0.005	0.942	35.924	<0.001	0.112	0.738
Proportion of False Alarms *N* = 1 & *N* = 2 Trials	0.103	0.011	0.113	0.010	0.073	0.011	0.077	0.010	0.277	0.600	37.423	<0.001	0.311	0.578
Omissions *N* = 1 & *N* = 2 Trials	4.941	0.951	4.135	0.911	3.265	0.951	2.230	0.911	0.685	0.409	6.431	0.012	0.026	0.871
Proportion Correct 0-Back Trials	0.922	0.009	0.942	0.009	0.930	0.009	0.921	0.009	0.272	0.603	0.675	0.413	4.109	0.045
Proportion Correct 0-Back Target Trials	0.832	0.022	0.877	0.021	0.826	0.022	0.813	0.021	0.412	0.522	4.514	0.035	3.151	0.078
Proportion Correct 0-Back Non-Target Trials	0.960	0.006	0.969	0.005	0.975	0.006	0.968	0.005	0.036	0.851	2.083	0.151	3.232	0.074
Proportion of False Alarms 0-Back Trials	0.027	0.004	0.020	0.004	0.016	0.004	0.022	0.004	0.011	0.916	1.584	0.210	3.905	0.050
Omissions 0-Back Trials	0.676	0.181	0.662	0.174	0.706	0.181	0.527	0.174	0.230	0.632	0.124	0.726	0.299	0.585
Proportion Correct 1-Back Trials	0.828	0.016	0.793	0.016	0.858	0.016	0.883	0.016	0.055	0.815	38.440	<0.001	9.446	0.003
Proportion Correct 1-Back Target Trials	0.663	0.037	0.595	0.035	0.693	0.037	0.743	0.035	0.036	0.850	17.047	<0.001	7.471	0.007
Proportion Correct 1-Back Non-Target Trials	0.899	0.014	0.879	0.013	0.929	0.014	0.943	0.013	0.036	0.849	27.748	<0.001	3.602	0.060
Proportion of False Alarms 1-Back Trials	0.074	0.011	0.081	0.011	0.051	0.011	0.048	0.011	0.020	0.887	20.358	<0.001	0.723	0.397
Omissions 1-Back Trials	1.456	0.414	2.176	0.397	1.294	0.414	0.419	0.397	0.030	0.864	7.373	0.007	5.096	0.026
Proportion Correct 2-Back Trials	0.702	0.015	0.701	0.014	0.749	0.015	0.744	0.014	0.026	0.872	21.931	<0.001	0.039	0.843
Proportion Correct 2-Back Target Trials	0.456	0.030	0.424	0.028	0.459	0.030	0.468	0.028	0.096	0.757	1.821	0.179	1.348	0.248
Proportion Correct 2-Back Non-Target Trials	0.807	0.017	0.819	0.016	0.873	0.017	0.862	0.016	0.001	0.970	20.329	<0.001	0.901	0.344
Proportion of False Alarms 2-Back Trials	0.133	0.012	0.146	0.012	0.095	0.012	0.106	0.012	0.678	0.412	25.732	<0.001	0.013	0.911
Omissions 2-Back Trials	3.485	0.684	1.959	0.655	1.971	0.684	1.811	0.655	1.135	0.288	2.552	0.112	1.721	0.192
Reaction Time														
*N* = 1 & *N* = 2 Correct Trials	556.709	12.135	560.564	11.633	489.173	12.135	504.664	11.633	0.405	0.525	73.644	<0.001	0.655	0.420
*N* = 1 & *N* = 2 Correct Target Trials	568.768	12.559	587.692	12.314	510.154	12.622	535.192	12.314	1.968	0.163	47.706	<0.001	0.144	0.705
*N* = 1 & *N* = 2 Correct Non-Target Trials	558.736	12.338	558.249	11.774	486.627	12.283	498.767	11.823	0.144	0.705	79.812	<0.001	0.735	0.393
0-Back Correct Trials	473.973	7.327	494.045	7.024	439.422	7.327	447.410	7.024	2.323	0.130	90.053	<0.001	1.995	0.160
0-Back Correct Target Trials	503.531	9.178	525.540	8.798	482.800	9.224	487.257	8.839	1.374	0.243	24.918	<0.001	2.204	0.140
0-Back Correct Non-Target Trials	463.660	7.571	484.093	7.257	424.510	7.571	433.331	7.257	2.411	0.123	95.073	<0.001	1.586	0.210
1-Back Correct Trials	540.297	11.420	543.248	10.947	471.964	11.420	493.422	10.947	0.750	0.388	67.624	<0.001	1.659	0.200
1-Back Correct Target Trials	549.380	12.953	569.375	12.532	495.606	12.953	530.893	12.286	3.108	0.080	28.011	<0.001	0.770	0.382
1-Back Correct Non-Target Trials	541.266	11.569	541.198	11.090	467.466	11.569	483.921	11.090	0.332	0.565	78.511	<0.001	1.248	0.266
2-Back Correct Trials	573.122	14.418	577.880	13.821	506.382	14.418	515.906	13.821	0.159	0.691	53.324	<0.001	0.073	0.787
2-Back Correct Target Trials	585.146	15.299	606.483	14.760	528.300	15.386	542.221	15.082	0.910	0.342	31.453	<0.001	0.118	0.732
2-Back Correct Non-Target Trials	574.825	14.678	575.301	14.004	505.789	14.609	514.069	14.065	0.058	0.809	51.092	<0.001	0.183	0.669

**Table 3:** Results of the acute LME for all dependent outcome measures for the Go/No-Go task.

	Pre-Adminstration				Post-Administration				Effect of Treatment		Effect of Time		Treatment Time	
	Placebo (*n* = 97)		CCE (*n* = 99)		Placebo		CCE							
	M	SE	M	SE	M	SE	M	SE	F (1, 194)	*p*	F (1, 194)	*p*	F (1, 194)	*p*
Accuracy														
Proportion Correct Overall	0.937	0.010	0.940	0.010	0.916	0.010	0.948	0.010	2.013	0.158	0.734	0.393	3.699	0.056
Hit Rate	0.965	0.011	0.965	0.011	0.945	0.011	0.979	0.011	1.658	0.199	0.098	0.754	4.417	0.037
Commission Rate	0.307	0.016	0.275	0.016	0.337	0.016	0.325	0.016	1.149	0.285	15.727	0.000	1.012	0.316
Reaction Time														
Mean Reaction Time Overall	444.211	6.127	435.977	6.065	416.403	6.127	413.853	6.065	0.448	0.504	66.149	0.000	0.857	0.356
Mean Hit Reaction Time	445.312	6.131	437.330	6.069	417.816	6.131	415.368	6.069	0.419	0.518	64.368	0.000	0.806	0.370
Mean Commission Rate	408.855	7.687	396.078	7.577	377.167	7.624	375.007	7.547	0.664	0.416	21.910	0.000	0.887	0.347

**Table 4:** Demographic information and statistics for participants included in both the *n*-back and Go/No-Go analyses for the longitudinal analyses.

	*N*-Back				Go/No-Go			
	Placebo (*n* = 92)	CCE (*n* = 96)	Statistic	*p*	Placebo (*n* = 73)	CCE (*n* = 81)	Statistic	*p*
Ethnicity								
American Indian or Alaskan Native, Hispanic or Latino, Asian or Asian American	1.09%	4.17%	χ^2^ = 9.644	0.210	1.37%	3.70%	χ^2^ = 6.195	0.517
Asian or Asian American	3.26%	3.13%			5.48%	3.70%		
Black of African American	2.17%	8.33%			2.74%	6.17%		
Hawaiian or Pacific Islander, White	0.00%	2.08%			0.00%	2.47%		
Hispanic or Latino	6.52%	6.25%			8.22%	6.17%		
White	85.87%	75.00%			80.82%	76.54%		
Mixed race	0.00%	0.00%			0.00%	0.00%		
Preferred not to say	1.09%	1.04%			1.37%	1.23%		
Education								
High School Diploma	3.26%	3.13%	χ^2^ = 4.392	0.734	4.11%	6.17%	χ^2^ = 4.987	0.662
Post-secondary Non-Degree Award	1.09%	0.00%			0.00%	0.00%		
Associate’s Degree	8.70%	10.42%			6.85%	11.11%		
Some College, No Degree	17.39%	12.50%			19.18%	18.52%		
Bachelor’s Degree	41.30%	38.54%			41.10%	30.86%		
Master’s Degree	19.57%	23.96%			21.92%	23.46%		
Doctoral or Professional Degree	8.70%	9.38%			6.85%	6.17%		
Other	0.00%	0.00%			0.00%	1.23%		
No Response	0.00%	2.08%			0.00%	2.47%		
Employment								
Homemaker	8.70%	3.13%	χ^2^ = 16.620	0.083	8.22%	4.94%	χ^2^ = 12.672	0.178
Student	0.00%	2.08%			0.00%	1.23%		
Employed for Wages	64.13%	66.67%			68.49%	59.26%		
Military	0.00%	1.04%			0.00%	1.23%		
Other	0.00%	1.04%			0.00%	0.00%		
Out of Work—Looking	4.35%	4.17%			4.11%	4.94%		
Out of Work—Not Looking	0.00%	3.13%			0.00%	3.70%		
Retired	10.87%	7.29%			6.85%	9.88%		
Self-Employed	8.70%	5.21%			6.85%	4.94%		
Unable to Work	3.26%	1.04%			5.48%	2.47%		
No Response	0.00%	5.21%			0.00%	7.41%		
Marital Status								
Divorced	10.87%	15.63%	χ^2^ = 9.752	0.135	10.96%	14.81%	χ^2^ = 10.829	0.094
Married or Domestic Partnership	68.48%	57.29%			72.60%	60.49%		
Other	1.09%	0.00%			1.37%	0.00%		
Separated	3.26%	1.04%			4.11%	2.47%		
Single, Never Married	15.22%	17.71%			10.96%	11.11%		
Widowed	1.09%	3.13%			0.00%	3.70%		
No Response	0.00%	5.21%			0.00%	7.41%		
Household Size								
1 Person	15.22%	20.83%	χ^2^ = 9.965	0.126	13.70%	19.75%	χ^2^ = 11.711	0.069
2 People	38.04%	32.29%			30.14%	30.86%		
3 People	17.39%	22.92%			17.81%	22.22%		
4 People	16.30%	8.33%			20.55%	8.64%		
5 People	8.70%	8.33%			10.96%	6.17%		
6 or More People	4.35%	2.08%			6.85%	4.94%		
No Response	0.00%	5.21%			0.00%	7.41%		
Household Income								
Less than $25,000	4.35%	4.17%	χ^2^ = 8.056	0.529	4.11%	6.17%	χ^2^ = 13.953	0.124
$25,000–$34,999	3.26%	5.21%			2.74%	6.17%		
$35,000–$49,999	7.61%	7.29%			6.85%	9.88%		
$50,000–$74,999	17.39%	16.67%			15.07%	14.81%		
$75,000–$99,999	22.83%	22.92%			20.55%	22.22%		
$100,000–$149,999	25.00%	21.88%			27.40%	18.52%		
$150,000–$199,999	6.52%	9.38%			10.96%	12.35%		
$200,000 or more	9.78%	4.17%			10.96%	2.47%		
I prefer not to say	3.26%	3.13%			1.37%	0.00%		
No Response	0.00%	5.21%			0.00%	7.41%		
Geographical Region								
Midwest—IA, IL, IN, KS, MI, MN, MO, ND, NE, OH, SD, WI	21.74%	21.88%	χ^2^ = 5.453	0.363	28.77%	22.22%	χ^2^ = 5.581	0.349
Northeast—CT, DC, DE, MA, MD, ME, NH, NJ, NY, PA, RI, VT	16.30%	12.50%			13.70%	11.11%		
Southeast—AL, AR, FL, GA, KY, LA, MS, NC, SC, TN, VA, WV	31.52%	36.46%			32.88%	33.33%		
Southwest—AZ, NM, OK, TX	14.13%	13.54%			10.96%	13.58%		
West—AK, CA, CO, HI, ID, MT, NV, OR, UT, WA, WY	16.30%	11.46%			13.70%	13.58%		
No Response	0.00%	4.17%			0.00%	6.17%		
Sex Assigned at Birth								
Male	33	38	χ^2^ = 0.276	0.600	25	32	χ^2^ = 0.456	0.500
Female	59	58			48	49		
Age (M ± SD)	49.61 ± 7.46	50.91 ± 7.63	F (1, 185) = 1.380	0.242	48.36 ± 6.82	51.09 ± 7.92	F (1, 151) = 5.182 *	0.024

* = Some participants declined to provide their specific age, and only responded to an age range question.

**Table 5:** Results from longitudinal LME modeling of the *n*-back task.

	Effect of Treatment				Effect of Time		Treatment Time		Main Effect of Time & Interaction	
	F	*p*	Numerator *df*	Denominator *df*	F	*p*	F	*p*	Numerator *df*	Denominator *df*
Accuracy										
Proportion Correct *N* = 1 & *N* = 2 Combined	2.129	0.146	1	201.068	23.708	<0.001	0.947	0.436	4	654.212
Proportion Correct *N* = 1 & *N* = 2 Target Trials	0.094	0.759	1	196.458	9.487	<0.001	2.235	0.064	4	665.183
Proportion Correct *N* = 1 & *N* = 2 Non-Target Trials	4.925	0.028	1	203.427	16.539	<0.001	0.118	0.976	4	619.148
Proportion of False Alarms *N* = 1 & *N* = 2 Trials	0.204	0.652	1	195.684	15.564	<0.001	0.136	0.969	4	641.268
Omissions *N* = 1 & *N* = 2 Trials	8.284	0.004	1	196.894	4.781	<0.001	0.086	0.987	4	585.205
Proportion Correct 0-Back Trials	2.760	0.098	1	221.430	1.766	0.134	0.425	0.791	4	612.745
Proportion Correct 0-Back Target Trials	1.859	0.174	1	218.398	0.955	0.432	0.530	0.714	4	619.311
Proportion Correct 0-Back Non-Target Trials	2.595	0.108	1	272.157	2.198	0.068	0.450	0.772	4	613.690
Proportion of False Alarms 0-Back Trials	1.725	0.190	1	301.950	1.525	0.193	1.523	0.194	4	634.316
Omissions 0-Back Trials	1.712	0.192	1	214.768	2.203	0.067	2.034	0.088	4	573.777
Proportion Correct 1-Back Trials	0.987	0.322	1	206.676	12.756	<0.001	2.112	0.078	4	640.984
Proportion Correct 1-Back Target Trials	0.260	0.611	1	195.324	6.908	<0.001	1.306	0.266	4	653.941
Proportion Correct 1-Back Non-Target Trials	1.805	0.180	1	229.925	10.032	<0.001	1.355	0.248	4	597.683
Proportion of False Alarms 1-Back Trials	0.320	0.572	1	196.861	8.678	<0.001	2.825	0.024	4	612.677
Omissions 1-Back Trials	2.193	0.140	1	264.435	2.956	0.019	1.135	0.339	4	592.520
Proportion Correct 2-Back Trials	3.088	0.080	1	200.852	18.621	<0.001	0.085	0.987	4	640.454
Proportion Correct 2-Back Target Trials	0.002	0.962	1	205.279	6.064	<0.001	1.710	0.146	4	656.353
Proportion Correct 2-Back Non-Target Trials	6.237	0.013	1	203.483	13.407	<0.001	0.577	0.679	4	621.606
Proportion of False Alarms 2-Back Trials	0.102	0.749	1	213.668	10.630	<0.001	1.116	0.348	4	637.502
Omissions 2-Back Trials	8.833	0.003	1	179.915	4.630	0.001	0.731	0.571	4	589.298
Reaction Time										
*N* = 1 & *N* = 2 Correct Trials	0.000	0.987	1	194.698	22.125	<.001	0.527	0.716	4	658.257
*N* = 1 & *N* = 2 Correct Target Trials	0.364	0.547	1	196.034	14.803	<.001	0.236	0.918	4	604.547
*N* = 1 & *N* = 2 Correct Non-Target Trials	0.001	0.971	1	193.120	24.577	<.001	0.498	0.737	4	654.277
0-Back Correct Trials	0.620	0.432	1	198.526	15.017	<0.001	0.175	0.951	4	649.023
0-Back Correct Target Trials	0.948	0.331	1	192.765	2.936	0.020	0.238	0.917	4	630.231
0-Back Correct Non-Target Trials	0.566	0.453	1	199.570	17.907	<0.001	0.252	0.909	4	644.080
1-Back Correct Trials	0.062	0.803	1	191.793	19.437	<0.001	0.713	0.583	4	650.419
1-Back Correct Target Trials	0.195	0.659	1	192.608	10.939	<0.001	0.443	0.777	4	617.025
1-Back Correct Non-Target Trials	0.050	0.824	1	191.700	20.789	<0.001	0.622	0.647	4	646.554
2-Back Correct Trials	0.023	0.881	1	198.949	16.211	<0.001	0.363	0.835	4	653.667
2-Back Correct Target Trials	0.162	0.688	1	201.529	8.674	<0.001	0.140	0.967	4	605.549
2-Back Correct Non-Target Trials	0.046	0.830	1	196.271	18.693	<0.001	0.369	0.831	4	650.948

**Table 6:** Results from longitudinal LME modeling of the Go/No-Go task.

	Effect of Treatment				Effect of Time		Treatment Time		Main Effect of Time & Interaction	
	F	*p*	Numerator *df*	Denominator *df*	F	*p*	F	*p*	Numerator *df*	Denominator *df*
Accuracy										
Proportion Correct Overall	0.120	0.730	1	158.482	2.125	0.077	1.486	0.205	4	487.369
Hit Rate	0.014	0.905	1	153.627	1.756	0.137	1.642	0.162	4	463.622
Commission Rate	0.702	0.403	1	172.693	1.261	0.284	0.680	0.606	4	535.317
Reaction Time										
Mean Reaction Time Overall	0.415	0.520	1	161.847	4.988	<0.001	0.695	0.596	4	547.083
Mean Hit Reaction Time	0.371	0.543	1	161.681	5.180	<0.001	0.664	0.617	4	547.055
Mean Commission Reaction Time	0.780	0.378	1	185.008	0.269	0.898	0.551	0.698	4	490.573

## Data Availability

The data presented in this study are available on request from the corresponding author due to privacy restrictions.
